# Endovascular treatment of arteriovenous graft dysfunction as a result of graft delamination and fracture

**DOI:** 10.1186/s42155-020-00181-8

**Published:** 2021-01-02

**Authors:** Aditi Chaurasia, Tushar Garg, Rakesh Ahuja, Michael Ferra

**Affiliations:** 1 Department of Radiodiagnosis, All India Institute of Medical Sciences Bhopal, Bhopal, India; 2 Department of Interventional Radiology, Seth GS Medical College & KEM Hospital, Mumbai, India; 3 Department of Radiology - Division of Vascular and Interventional Radiology, Einstein Medical Center, Philadelphia, USA

**Keywords:** Arteriovenous graft, Graft delamination, Dialysis intervention, Gore Acuseal

## Abstract

**Background:**

Graft thrombosis due to fabric delamination is a rare cause of delayed failure of arteriovenous grafts. Graft delamination is primarily an imaging diagnosis and is confirmed with the help of ultrasound which shows the separation of graft fabric layers. Only two such cases have been described in the literature so far.

**Case presentation:**

We present a case of upper extremity arteriovenous graft thrombosis in a 79 year old COVID-19 positive patient with end-stage renal disease. The diagnosis was established on ultrasonography which revealed separation of the graft fabric layers with thrombosis within the “false” and “true” lumen of the graft. The patient was managed with angioplasty and embolectomy of the clot material followed by stent-graft placement across the delaminated portion of the graft. Post-procedural angiography confirmed brisk flow across the graft and patient could successfully have subsequent hemodialysis sessions.

**Conclusions:**

Identification of graft delamination as a cause of graft failure is important as its management differs from other conventional causes since it requires stent-grafts to cover the area of delamination to re-establish flow and salvage the AV graft. The recognition of this phenomenon is essential to provide quality care and successful reuse of the AV graft.

**Level of evidence:**

Level 4, Case Report.

## Background

End-stage renal disease (ESRD) affects over 660,000 patients in the United States, including approximately 468,000 who require dialysis. Arteriovenous fistulae, arteriovenous grafts and central venous catheters are the three most common forms of vascular accesses for hemodialysis. An arteriovenous graft is a synthetic tubing, typically made of expanded PolyTetra-Fluoroethylene (ePTFE), which is interposed between an artery and vein. The graft is the site of needle access for dialysis. Delayed failure of AV grafts is most commonly due to stenosis or occlusion of the graft-vein anastomosis due to pressure from the arterialized blood being received by the elastic native venous system. However, only a few cases of graft thrombosis due to fabric delamination have been reported so far. In this article, we describe a case of graft thrombosis due to the rare phenomenon of fabric delamination and its management.

## Case presentation

**Fig. 1:**
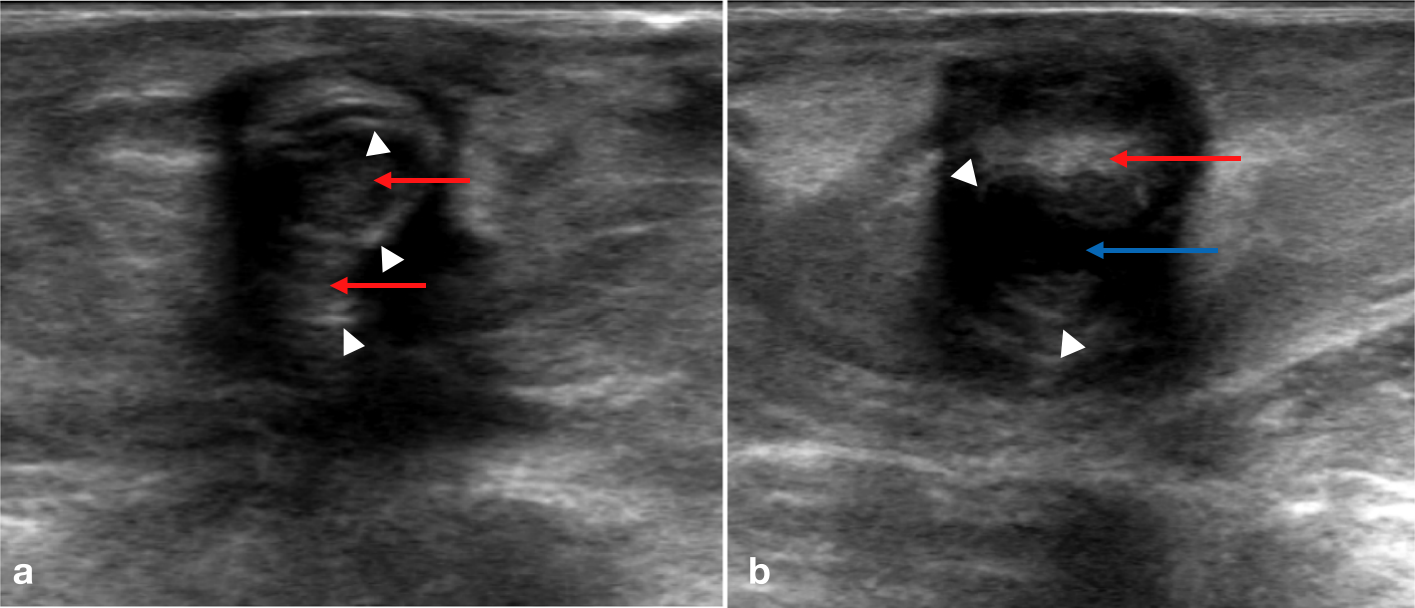
**a, b** Doppler ultrasonography of left brachio-axillary arteriovenous graft showing echogenic linear material floating distinctly within the graft lumen (blue arrow), suggestive of delaminated graft fabric layers (arrowheads). It also shows thrombus in the false lumen created by graft delamination and an intraluminal thrombus (red arrows) (**a**)

**Fig. 2:**
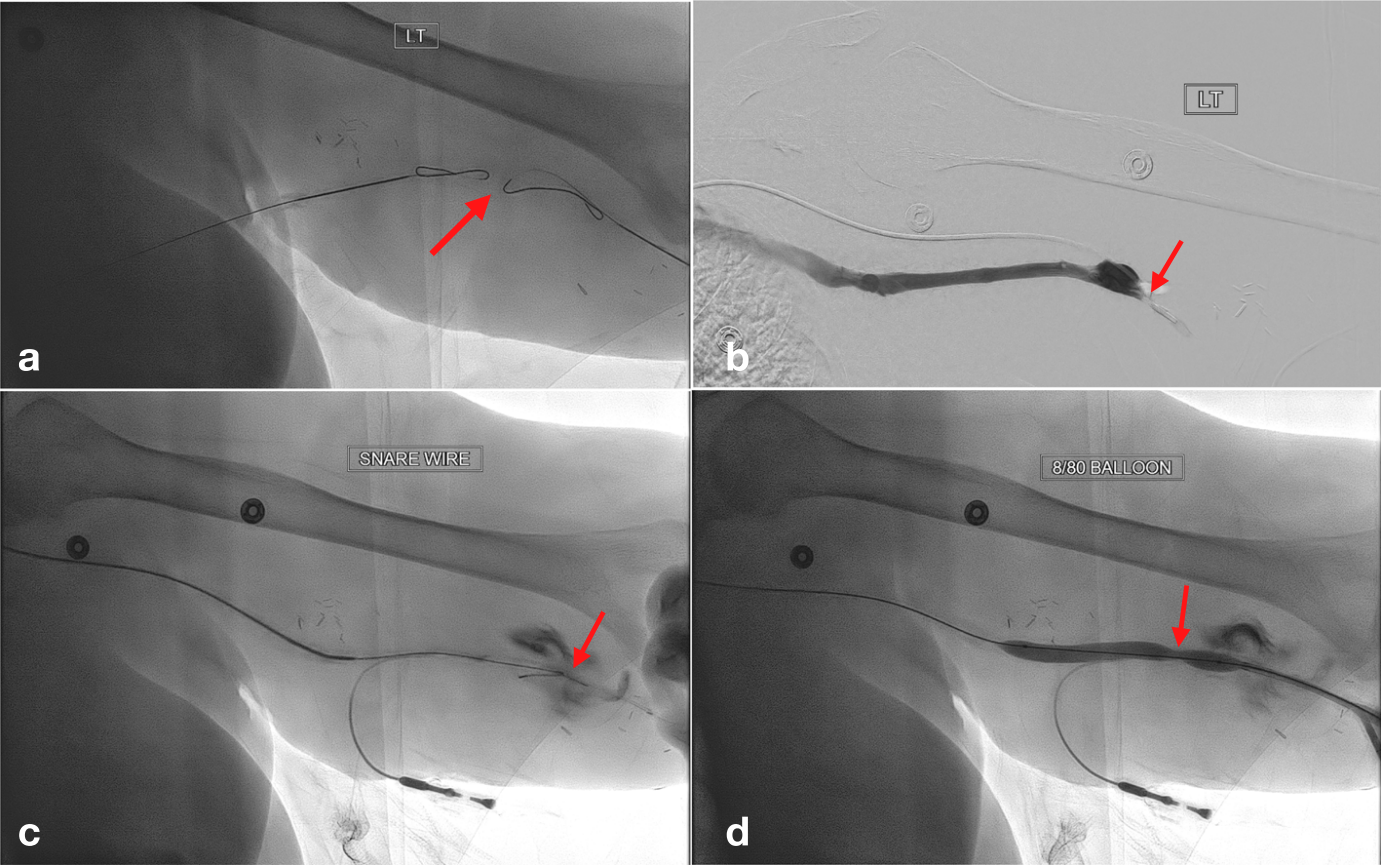
Failure to advance a 018″ guidewire viz. local antegrade and retrograde accesses due to focal obstruction (arrow) in the midportion of the graft correlating with area of delamination seen on ultrasound (**a**). Transfemoral access used to selectively catheterize the left brachial vein and contrast injection confirmed position within the graft vein anastomosis (arrow) (**b**). Four to eight millimeters microsnare via antegrade micropuncture sheath used to externalize the glidewire from the arm creating a through and through wire access (arrow) (**c**). Persistent flow defect is seen in the mid-portion of the graft (arrow) after Balloon angioplasty and platelet plug embolectomy of the graft-artery anastomosis demonstrating the area of delamination which needs to be grafted (**d**)

A 79-year-old woman with ESRD and a positive COVID-19 test on maintenance hemodialysis presented to the emergency department after multiple failed attempts to cannulate her left upper extremity AV graft. The patient had potassium of 5.2 on admission and did not have any signs of volume overload or uremia to suggest the need for urgent hemodialysis. Physical examination of the left upper extremity was significant for the absence of any thrill or pulsatility along the course of the AV graft suggestive of graft thrombosis. Given the patient’s COVID-19 positive status, the decision was made to admit the patient in observation status and perform a graft thrombectomy. Due to the patient’s contrast allergy, an overnight 13-h steroid regimen was administered and the procedure was performed the following morning. The patient was brought to the IR suite and the left arm was prepped and draped in a sterile fashion. A limited ultrasound of the left arm AV graft showed separation of the graft fabric layers with thrombus in the false lumen between the superficial fabric layer and delaminated layer as well as a thrombus in the “true” lumen between the delaminated layer and the deep fabric layer (Fig. 1a, b). Under the fluoroscopic guidance, a micropuncture needle was inserted into the graft in an antegrade fashion near the graft artery anastomosis and a 0.018″ wire was advanced through the needle but was unable to be advanced beyond the midportion of the graft. Ultrasound-guided micropuncture needle access was obtained in a retrograde fashion, however again an 0.018″ wire was unable to be advanced beyond the midportion of the graft (Fig. 2a). This occurred due to a micropuncture sheath being within the delaminated portion of the graft instead of the proper lumen.

The micropuncture needles were exchanged over the 0.018″ wires for micropuncture upsizing sheaths. Attempts were made to advance 0.035″ glide wires (Terumo Corp; Tokyo, Japan) without success. The contrast was then injected from the antegrade and retrograde upsizing sheaths demonstrating access within the delaminated false lumen which showed a thin “tail” of contrast emptying into a widely patent native axillary vein.

Transfemoral access was then obtained via the left common femoral vein and an 8 Fr sheath was placed. A 5 Fr angled catheter was advanced over a glide wire (Terumo Corp; Tokyo, Japan) and was used to cross the graft vein anastomosis and gain access into the true lumen of the graft. A contrast injection demonstrated patency of the graft beyond the area of delamination without evidence of venous anastomosis stenosis (Fig. 2b).

A 3.2 Fr 4–8 mm En-Snare (Merit Medical; South Jordan, Utah) was advanced through the antegrade upsizing sheath and was used to capture the glide wire providing through and through access in the true lumen (Fig. 2c). The upsizing sheath was exchanged over the wire for a 7Fr sheath and balloon angioplasty of the graft was performed using an 8 mm Mustang angioplasty balloon (Boston Scientific, Marlborough, MA) showing a recurrent moderate to severe waist in the midportion of the graft (Fig. 2d).

**Fig. 3:**
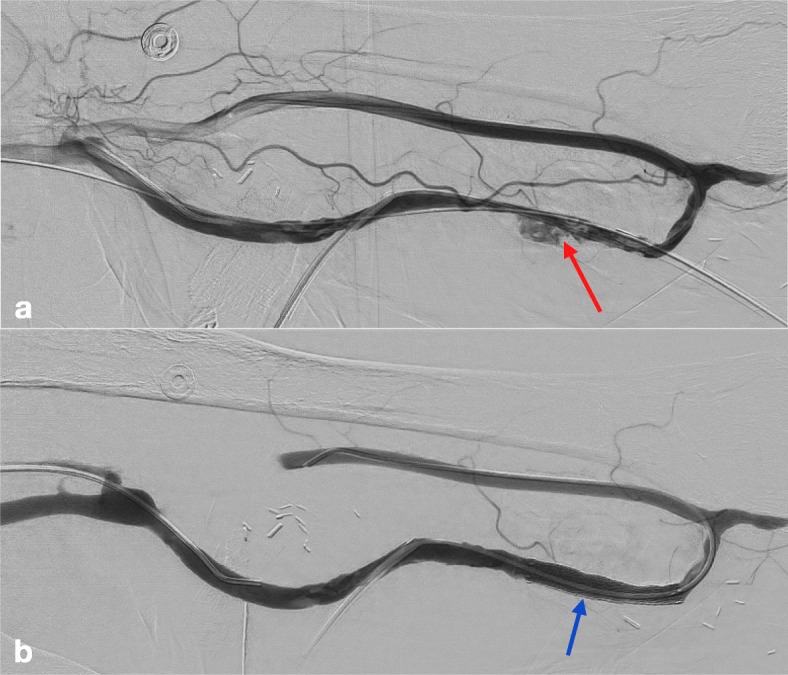
Focal irregularity (red arrow) at the site of delamination, did not expand to suggest true extravasation (**a**) Final angiogram after placement of covered stent graft (Viabahn Endoprosthesis) (blue arrow) across the site of delamination showing correction of the focal irregularity and improved flow across the fistula (**b**)

Under ultrasound guidance, the graft’s true lumen was then re-accessed in a retrograde fashion and a 6 Fr sheath was placed. Balloon angioplasty and platelet plug embolectomy of the graft artery anastomosis was performed with a 5 mm Mustang angioplasty balloon (Boston Scientific, Marlborough, MA). A brachial arteriogram was then performed which showed restoration of arterial flow through the graft. A focal area of extra-luminal contrast was identified (Fig. 3a) correlating to the area of fabric delamination. Via the retrograde access, an 8 mm × 50 mm Gore Viabahn Endoprosthesis (Gore Medical; Newar, DE) was deployed across the area of delamination and post dilated with an 8 mm angioplasty balloon. A completion brachial arteriogram demonstrated brisk flow through the graft with the resolution of the previously seen area of delamination (Fig. 3b).

Since the completion of the procedure, the patient has undergone 36 successful hemodialysis sessions, which signifies that the fistula has been successfully salvaged.

## Conclusions

Numerous reports of graft delamination have been published in the US Food and Drug Administration’s Manufacturer and User Facility Device Experience Database, but only two cases have been published to date, which reports endovascular management of this complication (Dai et al. 2020). In our case, there was no significant stenosis at the venous anastomosis, but there was presence of thrombus in the entirety of the graft, which was partially corrected with balloon angioplasty and balloon-assisted platelet plug embolectomy. Interestingly, there was a persistent defect in the midportion of the graft appearing distinct from the typical intra-graft concentric stenosis and intraluminal thrombus. This occurred due to the delamination of the graft which created a false lumen that was compressing the true lumen. To correct this delamination, after the failure of angioplasty, a Viabahn endoprosthesis was deployed to obtain normal, brisk flow through the graft.

Femoral access was used in our case to cannulate the graft lumen after the failure to access it through local antegrade and retrograde access. Transfemoral access was able to provide entry into the native graft lumen via the native venous system allowing wires and catheters to pass while avoiding the false lumen (Wang et al. 2019).

In patients with arteriovenous grafts, any process which disrupts the luminal surface of the graft such as hemodialysis needle cannulation, insertion of vascular sheaths for endovascular intervention or angioplasty or rotational thrombectomy can create a plane between the layers of the graft which allows the blood to enter the false lumen and result in further delamination of the graft material. Alternatively, there can also be delamination of the pseudointima, which is formed by a complex matrix of cells and fibroblasts over the graft luminal surface, and this can create a false lumen (Roy-Chaudhury et al. 2001). A manufacturing defect in the layer adherence or apposition can also predispose a graft to delamination in the future. In this case, the patient’s Gore Acuseal graft (Gore Medical; Newark, DE) is comprised of three layers (an abluminal ePTFE layer, an elastomeric layer, and a luminal heparin-coated ePTFE layer), which may have got separated causing the delamination, likely from repeated puncturing at the same spot during dialysis access from 17G needle.

Management of graft delamination differs from that of conventional stenosis as delamination cannot be corrected by conventional angioplasty and thrombectomy, which are typically used to salvage the AV grafts. In these patients, stent-grafts are needed to cover the area of delamination to open the true lumen and prevent the flow of blood into the false lumen. This can then be confirmed by improved flow visualization, physical examination and angiographic appearance. The recognition of this phenomenon is essential to provide quality care and successful reuse of the AV graft.

## Data Availability

Data sharing is not applicable to this article as no datasets were generated or analysed during the current study.

## Abbreviations

AV: Arteriovenous
ePTFE: Expanded PolyTetra-Fluoroethylene
IR: Interventional Radiology
